# A Study on the Pressure Mechanism Improvement of a Roller-Type Machine Working Bodies

**DOI:** 10.3390/ma16051956

**Published:** 2023-02-27

**Authors:** Auezhan T. Amanov, Gayrat A. Bahadirov, Ayder M. Nabiev

**Affiliations:** 1 Department of Mechanical Engineering, Sun Moon University, Asan 31460, Republic of Korea; 2 Tashkent Institute of Irrigation and Agricultural Mechanization Engineers, Tashkent 100000, Uzbekistan; 3 Institute of Mechanics and Seismic Stability of Structures, Academy of Sciences of the Republic of Uzbekistan, Tashkent 100125, Uzbekistan

**Keywords:** roller machine, pressure mechanism, working rolls, processed material, leverage

## Abstract

The parameters of the improved design of the pressure mechanism of a roller technological machine for squeezing wet materials are investigated in this article. The factors influencing the parameters of the pressure mechanism, which provide the required force between the working rolls of a technological machine during the processing of moisture-saturated fibrous materials, such as wet leather, were studied. The processed material is drawn in the vertical direction between the working rolls under their pressure. This study aimed to determine the parameters that make it possible to create the required pressure of the working rolls depending on the change in the thickness of the material being processed. A pressure mechanism of working rolls mounted on levers is proposed. In the design of the proposed device, the length of the levers does not change due to the movement of the sliders when turning the levers; this provides a horizontal direction of the sliders. The change in the pressure force of the working rolls is determined depending on the variation in the nip angle, the coefficient of friction, and other factors. Based on theoretical studies concerning the feed of the semi-finished leather product between the squeezing rolls, graphs were plotted, and conclusions were drawn. An experimental roller stand designated for pressing multi-layer leather semi-finished products has been developed and manufactured. An experiment was carried out to determine the factors affecting the technological process of squeezing excess moisture from wet semi-finished leather products with their multilayer package together with moisture-removing materials by means of their vertical supply on a base plate between rotating squeezing shafts also covered with moisture-removing materials. According to the results of the experiment, the optimal process parameters were selected. It is recommended to carry out the process of squeezing the moisture from two wet semi-finished leather products at a pass rate more than twice as high and with a pressing force of the working shafts two times lower compared to the analog. According to the results of the study, the optimal parameters for the process of squeezing the moisture from two layers of wet leather semi-finished products were chosen, namely the feed rate of 0.34 m/s and a pressing force of the squeezing rollers of 32 kN/m. The use of the proposed roller device allowed an increase of two times or more in the productivity of the process of processing wet leather semi-finished products on the basis of the proposed technique compared to known roller wringers.

## 1. Introduction

Scientific research is being conducted worldwide, and specific results are achieved in developing high-performance equipment and technologies. Several methods have been considered to reduce labor and energy costs and increase the efficiency of technological operations in processing local leather raw materials [1].

After liquid operations, semi-finished leather products contain more than 70% moisture. Water is squeezed out mechanically to perform further mechanical operations and reduce energy consumption. It has been experimentally determined earlier that the moisture content should be approximately 45–60% depending on the type of semi-finished leather product for normal mechanical operations [2]. Providing a moisture content of 45–60% is realized mechanically, in particular, through roller pressing.

Many shortcomings leading to defects in the semi-finished leather product during the mechanical processing of topographic sections of the hide, at uneven moisture content within 55–60%, have been listed in a previous study [3]. In the existing squeezing roller machines, the pressing devices do not provide the required pressing force when the thickness of the semi-finished leather product varies because the specific force increases. After squeezing, the defect appears in the form of folds that are not smoothed out. The pressing mechanisms of fleshing machines designed for low pressing forces when planning the fleshing have been suggested earlier [3,4,5,6]. However, they are unsuitable for pressing machines to extract moisture from semi-finished wet leather products.

Therefore, the improvement of existing designs and the introduction of new technological equipment is one of the promising areas for the innovative development of enterprises (manufacturing finished leather products), adapted to modern consumer requirements, ensuring the competitiveness of leather goods and the profitability of enterprises in domestic and foreign markets. Improving the quality of treating raw hides at all processing stages is necessary to solve these issues. Currently, scientists are researching new scientific and technical solutions for the creation of highly-efficient roller technological machines for processing semi-finished leather products. The influence of factors on the specific pressure in the roller nip of the module has been analyzed in a previous study [7]. As a result of experimental studies, the graphs of these dependencies were plotted, and a mathematical model was built. Short-term perturbations arising in roll pairs created by forced vibrations of the system have also been studied [8]. The authors of this work argued that the pattern of change in oscillations is affected by the form of their disturbance, the rate, and the properties of the processed material. Knowing such parameters makes it possible to solve the equation of forced oscillations of the working rolls.

China et al. analyzed the current state and methods to improve the efficiency of tanneries in Tanzania [9]. The authors indicated that along with many factors, it was revealed that certain factors hindering the prosperity of the industry are affected by the short introduction of new technologies and innovative developments in the production cycles of hiding processing. The nonlinear tension of materials characterized by increased tangential modulus with increasing load is studied [10]. The fibrous structure and the wavy shape of fibers explain the nonlinearity of tension. In the process of straightening the fibers, a geometric nonlinearity is associated with a sharp increase in resistance. Kuznetsov et al. developed a dynamic model of a deformable elastic–viscous body, considering the “unidirectional” viscosity [11]. In their model, contact at a certain point is maintained both under compression and reverse motion. However, the interaction force at this point under reverse motion is characterized only by rigidity. Viscous resistance did not occur under reverse motion. The study of the body strain process using this model was performed under the condition that the initial load provides a strain that exceeds the probable oscillation amplitude. The coefficients of static friction and sliding of various types of coatings of working rolls on metal were experimentally determined [12]. Dependences of the coefficient of sliding friction of the working rolls on the frequency of their rotation and friction were determined. Based on an in-depth study of numerous scientific publications devoted to processing hides, the authors identified factors that affect the increase in labor productivity in the leather industry in India [13]. Four factors were identified and analyzed in the study: personnel to perform processing, processing machine, processing method, and processed material. Various designs of rolls subjected to breaks and loading conditions were studied earlier [14]. The researchers conducted an in-depth analysis of existing methods of rolls and studied the causes of their fatigue and breakdown. Studies on the design and calculation of rolls were analyzed in detail. Some shortcomings in the roll design were found.

The problem of limit deflections of rolls caused by load and vibration as a function of the speed of roll rotation was studied [15]. It was revealed that non-proportional loads on the rolls of special machines significantly affect the operating modes and lifetime of machines. Based on experimental measurements, a measuring system of the forces arising at the points of contact of the conveyor belt with the pressure rollers was developed [16]. The authors stated that the process of transporting bulk materials was not fulfilled under the insufficient force of pressing rolls on the conveyor belt. This system allows the construction of regression models to analyze conveyor failures. A dynamic model for studying the vibrational characteristics of the roll–disk system was also developed [17]. The adequacy of the obtained model was verified based on simulation results. The influence of boundary conditions on the frequency and geometrical parameters of the roll–disk system was determined. A system for dynamic measurement of rotating roll vibration was developed [18]. The developed method provides a multidimensional measure of the vibration parameters of rotating rolls. The advantage of the proposed measurement system, which provides accuracy in determining the dynamic characteristics and analysis of rotating rolls in operating modes, was demonstrated. The two-decade research devoted to defining and analyzing roll breakage was studied and analyzed in detail. The authors proposed a system analysis mechanism for detecting roll failures under operating conditions. It was revealed that the breakage of rolls is mainly affected by wear, corrosion, deformation, and fatigue failure, due to periodic loads under their operation [19]. The effect of rolls on the vibration of a pair of tooth gears used in one and two rolls was studied [20]. The authors derived the equations of motion that determine the circumferential vibrations of a pair of tooth gears and torsional vibrations of rolls and performed numerical calculations. The design of the pressure mechanism of a roller technological machine for leather processing was studied [21]. The authors analytically determined the relationship between the pressing force on the drive lever pedal and the required pressure exerted on the cantilever working body. Equations were derived for determining the parameters of the driving foot pedal installation of a roller technological machine. In recent years, the authors have carefully analyzed the development trends of textile and leather equipment and devices. There has been a trend in the development and production of compact and efficient technological machines for the processing of hides and skins. Therefore, our research is aimed at achieving these goals. In addition, based on the results of the tests and experimental studies, improvements are gradually made to the design of the roll technological machines we are developing. For instance, the designs of the working bodies and the main actuators are being improved, taking into account the continuity of these mechanisms and parts. Based on a thorough study and analysis of the modern development of the design of roller technological machines for processing raw hides, we have developed new designs of roller devices for the mechanical processing of leather semi-finished products. New technical solutions are patented inventions [22,23]. These machines are suitable for use in leather production in small batches. On the basis of patents, we have manufactured experimental stands for roller devices. In addition, on these stands, we experimentally determined the deformation properties of the semi-finished leather product in its main topographic areas, harvested in the regions of Uzbekistan, as well as the cloth coverings of the pressing rolls [24].

## 2. The Design and Principle of Operation of the Mechanism

**Figure 1:**
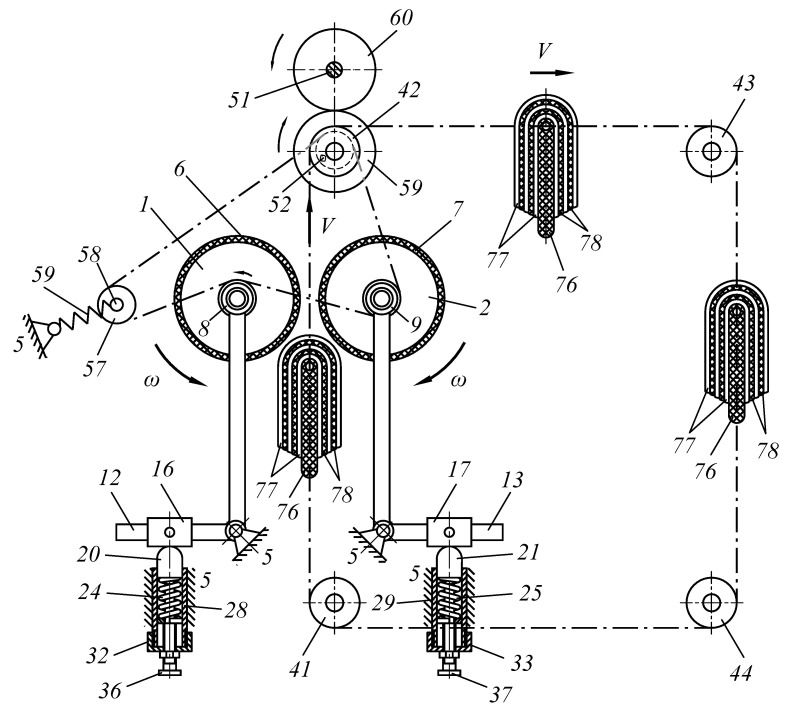
Scheme of a roller squeezing machine.

**Figure 2:**
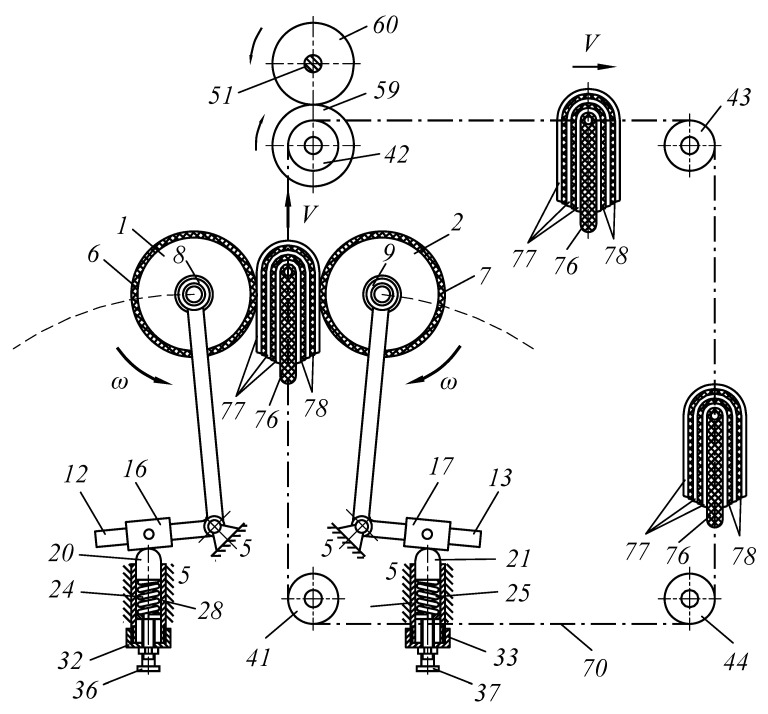
Scheme of motion of working bodies in contact with a package of processed leather semi-finished products.

**Figure 3:**
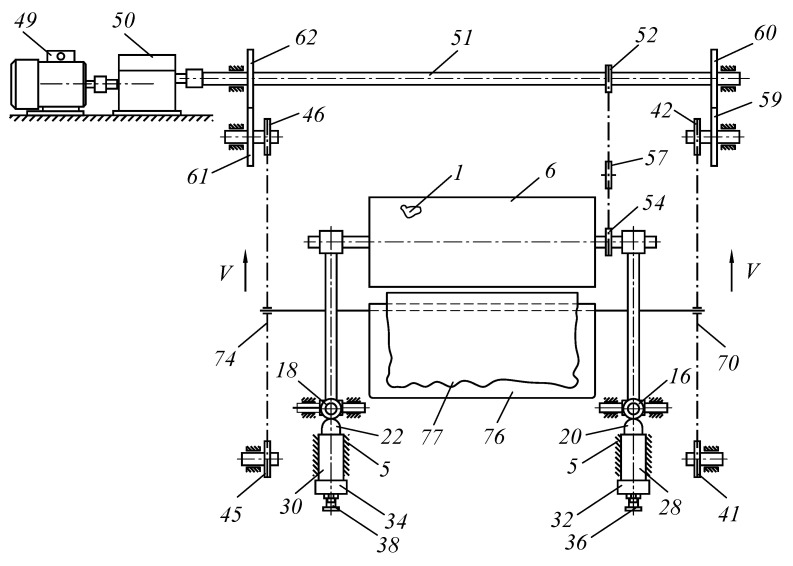
The scheme of transportation of the processed material by the working rolls in contact with the package of processed leather semi-finished products.

**Figure 4:**
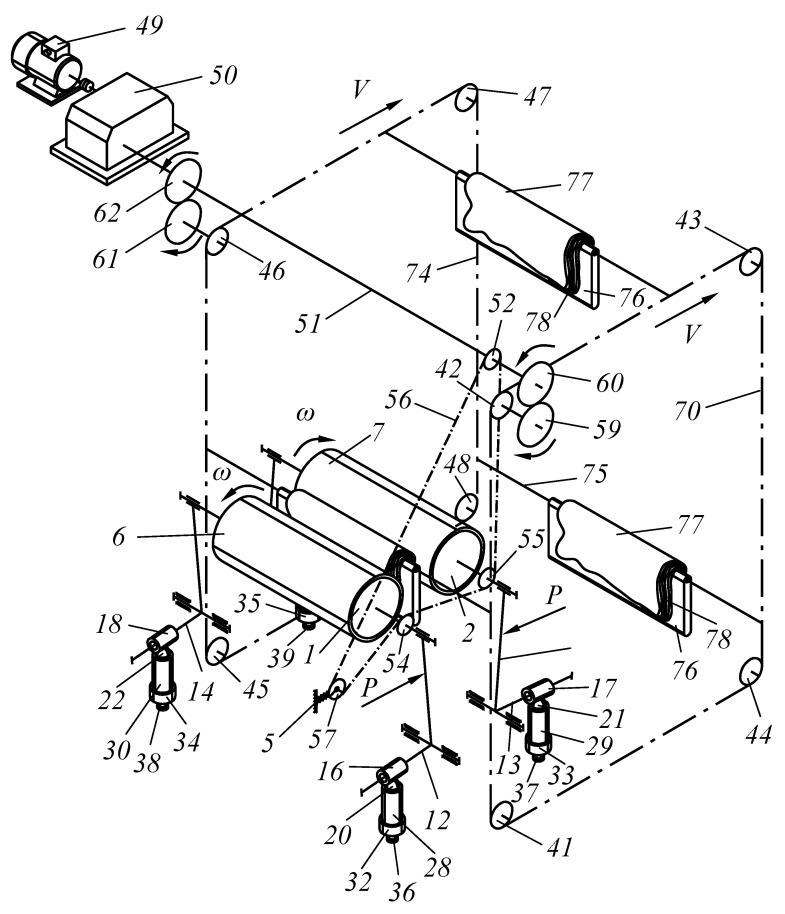
Scheme of the general view of the roller squeezing machine.

The purpose of the study was to develop a new technical solution that ensures the creation of the required pressing force between the working bodies of roller technological machines. In particular, to improve the design of roller machines, we proposed an improved pressure mechanism for working rolls to be used in the design of a roller squeezing machine. Moreover, the object of study is a pressing device that provides the required force between the working rolls of the squeezing machine when extracting moisture from wet semi-finished leather products. The methods of the theory of machines and mechanisms and analytical geometry were applied as research methods. A device was developed to provide the required force between the working bodies of specialized roll machines (Figure 1, Figure 2, Figure 3 and Figure 4).

To improve the quality of squeezing, it is necessary to provide the required pressing force of the working rolls by installing a feedback mechanism to the pressure control mechanism.

A double-arm lever, parallel to another, is installed in the device. Sliders are installed at the ends of these levers; the slider on the shoulder of one lever is connected to the slider on the shoulder of the next lever using a pipe and forks. The next slider on the other arm of the lever is connected to the slider on the next lever using forks, a cover, a glass, a rod, a piston, and an elastic element.

The feedback mechanism ensures the required pressing force acting at an increase in the thickness of the package of wet leather semi-finished products with moisture-removing materials; additional force is transferred to the supports of the working rolls, directed opposite to compression, with constant arms of double-arm levers.

In the design of the proposed device, the length of the double-arm levers does not change due to the movement of the sliders when the levers rotate, providing a horizontal direction of the sliders. Due to the installation of identical or different (or their combination) elastic elements (cylindrical or conical springs) of the working roll pressing mechanism and the feedback mechanism and their identical tightening, the required pressing force of the working rolls is ensured when the thickness of processed wet leather semi-finished products varies.

A new design of the roller machine is proposed; it contains squeezing rolls 1 and 2, mounted on bearings 3 and 4 on frame 5, and the rolls are located horizontally. Coatings 6 and 7 made of fibrous materials are put on the surface of the squeezing rollers (see Figure 1).

Double-arm levers 12, 13, 14, and 15 are mounted on bearings 8, 9, 10, and 11 on the trunnions of squeezing shafts 1 and 2, and one arm is located at a right angle relative to the other arm. These double-arm levers at the junction are pivotally located on frame 5. Sliders 16, 17, 18, and 19 are installed on the small arms of levers 12, 13, 14, and 15 (see Figure 2).

Supports 20, 21, 22, 23 resting on springs 24, 25, 26, 27, or on the rods of hydraulic cylinders are located under the sliders, and springs 24, 25, 26, and 27 are installed inside cylinders 28, 29, 30, 31. At the lower end of hydraulic cylinders 28, 29, 30, and 31, covers 32, 33, 34, and 35, are threaded. Bolts 36, 37, 38, and 39 are threaded in the center of covers 32, 33, 34, and 35 to tighten springs 24, 25, 26, and 27.

Vertical feed on base plate 40 is realized through two parallel chain conveyors 70 and 74, installed in the zones of the trunnions of squeezing rolls 1 and 2 on toothed sprockets 41, 42, 43, 44 and 45, 46, 47, 48, respectively. The rotation of squeezing rolls 1 and 2 is realized via electric motor 49 through gearbox 50 and shaft 51. Sprocket 52 is installed on shaft 51, and sprockets 54 and 55 are installed on squeezing rolls 1 and 2, respectively (Figure 3).

To tension chain 56, sprocket57 is installed on axle 58, placed on the fork with spring 59, and its other end is attached to frame 5. Gears 59 and 60 are installed on shaft 51 at both ends of squeezing rolls 1 and 2. Gears 59 and 60 are in contact with gears 61 and 62, respectively. Gears 61 and 62 are placed on axles 63 and 64, and sprockets 65 and 66 are installed at the other end. Chain 70 is mounted on sprockets 65, 67, 68, and 69. Chain 74 is mounted on sprockets 66, 71, 72, and 73. Rod 75 is mounted on chains 70 and 74, on which base plate 76 of fibrous permeable materials 78 is fixed. Semi-finished leather product 77 is bent over base plate 76, and a fibrous material of the LASCH type 78 is then installed, and so forth (Figure 4).

The roller machine works as follows. We turn on electric motor 49. The rotation is transmitted to gearbox 50 and then transferred to shaft 51. From it, the rotation through sprocket 52 is transmitted by chain 56 to sprockets 53, 54 and 55, installed on the squeezing rolls1 and 2, respectively. From shaft 51 through gear 59, rotation is transmitted to gear 61, which is mounted on axle 63. Then, from gear 66 on the shaft, rotation is transmitted through chain 74 to sprockets 71, 72, and 73. From shaft 51 through gear 60, rotation is transmitted to gear 62, mounted on axle 64.

Sprocket 65 is also installed on axle 64; it transmits the rotation through chain 70 to sprockets 67, 68, and 69. Rods 75 with base plate 76 are installed on chains 70 and 74, on which semi-finished leather product 77 with layers of moisture-moving material—monchon—78 lays.

Chains 70 and 74 transfer base plate 76 with leather semi-finished product 77 and moisture-removing material 78 to the space between rolls 1 and 2. In this case, levers 12, 13, 14, and 15 rotate around their axis, which leads to the compression of the springs via the small arms of the double-arm *L*-shaped lever and using sliders 16, 17, 18, and 19.

The deformation depends on the thickness of the package of base plate 76 with the leather semi-finished product 77 and moisture-removing material 78. Here, the pressing force is controlled by the thickness of the package, which significantly improves the quality of the process of squeezing wet leather semi-finished products. This is reflected dramatically in the multi-layer extraction of moisture from several layers of semi-finished leather products since it is not necessary to regulate the constancy of the pressing force of the working rolls depending on the thickness of the processed package of wet leather semi-finished products with monchons.

**Figure 5:**
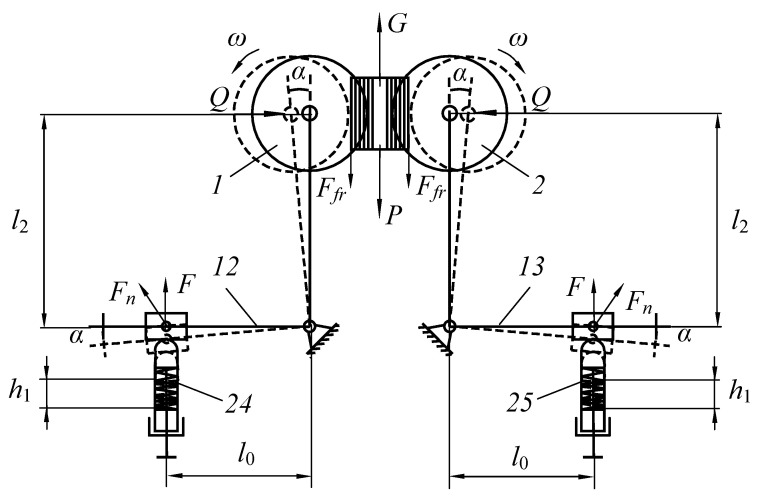
Scheme for calculating the parameters of the pressure mechanism of the roller machine.

Let us consider a particular case when processed material 3 came into contact with working rolls 1 and 2 (Figure 5). From Figure 5, we find the projection of acting forces onto the coordinate *oy* an *x* is.

(1) $$\sum F_{y}=0,G-P+2F=0$$

where *P* is the weight of the material being processed, *G* is the pulling force of chains, and *F* is the pressure force of springs.

From Figure 5, we determined the force of the moment relative to the points of initial contact and obtained the following:

(2) $$\sum M_{A}=0;\;\;\;\;F\cdot l_{2}+Q\cdot l_{0}=0.$$

From Expression (2), we determine the pressure force of the working rolls and obtain the following:

(3) $$Q=-\frac{F\cdot l_{2}}{l_{0}}$$

where F=Δh⋅c,$\Delta h=h_{0}-h_{1}$. c− is the stiffness of springs 24 and 25.

The spring pressure force according to (2) equals:

(4) $$F=-\frac{Q\cdot l_{0}}{l_{2}}$$

With Expression (4), the equilibrium Equation (1) can be written in the following form:

(5) $$G-P-2\left(\frac{Q\cdot l_{0}}{l_{2}}\right)=0$$

Thus, using Equation (5), we determine the pressure force of the working rolls.

(6) $$Q=\frac{l_{2}\left(G-P\right)}{2l_{0}}$$

Now consider the following special case, when the processed material is clamped between the working rolls under pressure. Figure 5 shows the projection of forces onto the coordinate *oy* and *x* is when the working rolls grip the material.

(7) $$\sum F_{y}=0,G-P+2F\sin\alpha -2F_{fr}\cos\alpha +2N\sin\alpha +2Q\cos\alpha =0$$

From Figure 5, we determine the force of the moment relative to the grip points and obtain the following expression:

(8) $${\sum M}_{B}=0;\;\;\;F_{n}\cdot l_{2}+Q\cdot l_{0}\cos\alpha =0.$$

where $F_{n}=F\sin\alpha$.

Therefore, using Expression (2), the pressure force of the springs is:

(9) $$F=-\frac{Q\cdot l_{0}\cos\alpha }{l_{2}\sin\alpha }$$

Substituting Equation(9) into the equilibrium Equation (7), we write an equation for determining the pressure force of the working rolls.

(10) $$G-P-2\left(\frac{Q\cdot l_{0}\cos\alpha }{l_{2}\sin\alpha }\right)\sin\alpha -2F_{fr}\cos\alpha +2N\sin\alpha +2Q\cos\alpha =0$$

Simplifying Equation (10), we determine the pressure force of the working rolls acting on the processed material from the resulting expression.

(11) $$Q=\frac{G-P+2N(\sin\alpha -f\cos\alpha )}{2\left(\frac{l_{0}}{l_{2}}-1\right)\cos\alpha }$$

Thus, the pressure force exerted on the base plate between working shafts 1 and 2 (Figure 5) can be expressed as the following expression:

$$F=c(h_{0}-h_{1})\frac{l_{0}}{l_{2}}\cos\alpha ;l_{2}>l_{0}.$$

where *c* is the specific pressure force of the elastic element; *(h*_0_ – *h*_1_*)* is the magnitude of the deformation, the ratio of the arms of the *L*-shaped lever is *l*_0_/*l*_2_, and the angle of inclination of the levers is cosα.

Thus, Equation (11) is obtained to determine the pressure force using *L*-shaped levers at different lengths of levers. By varying the length of the levers of the pressure mechanism of the working rolls, the required pressure is provided for compressing the material being processed between the roller pair.

## 3. Results and Discussion

Using the calculation scheme shown in Figure 5 and based on Equation (11), we plot graphs for special cases, for example, when the material being processed is clamped between the working rolls under pressure.

**Figure 6:**
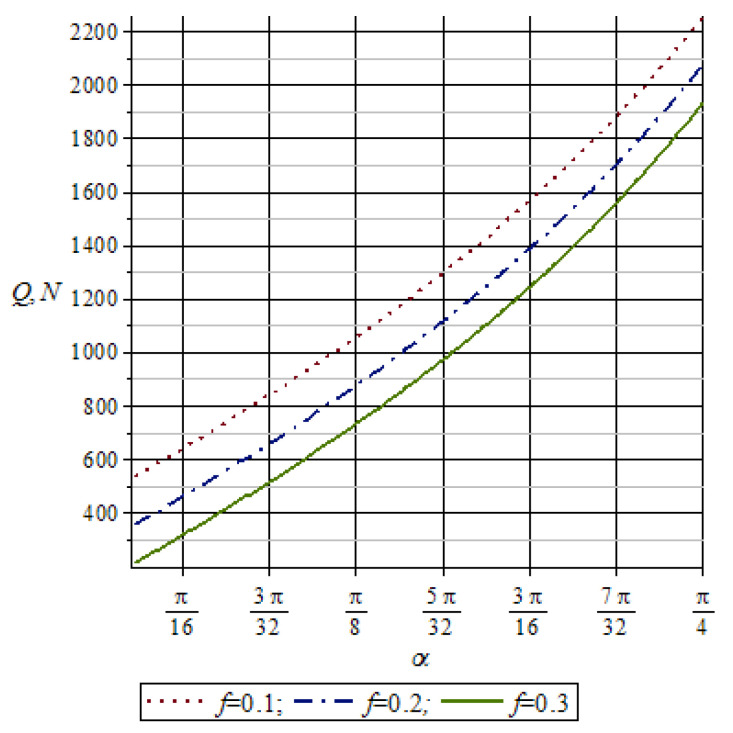
Graph of changes in the pressure force of the working rolls depending on the difference in the nip angle.

Figure 6 shows a diagram of the dependence of the pressure force of the working rolls *Q* on the nip angle *α*, taking into account the variation in the friction coefficient for *f* = 0.1; *f* = 0.2; *f* = 0.3 and the change in the nip angle from 0 to 45 degrees.

An analysis of the graphs in Figure 6 showed that with an increase in the nip angle α, the pressure force of the working rolls *Q* increases, and at low friction coefficient *f*, the pressure force of the working rolls *Q* increases.

**Figure 7:**
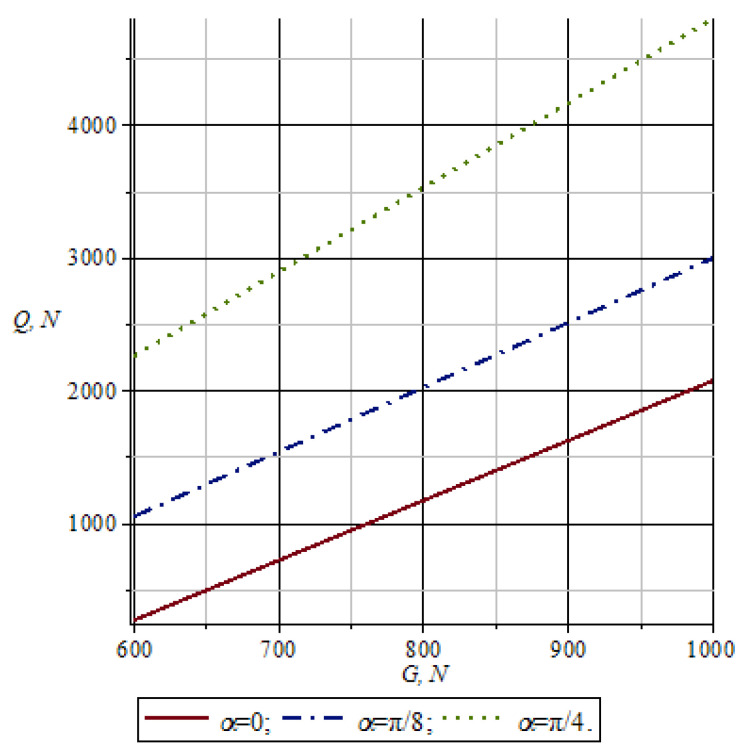
Graph of changes in the pressure force of the working rolls depending on the pulling force of conveyor chains.

Figure 7 shows the changes in the pressure of working rolls *Q* depending on the pull force *G* of conveyor chains with a shift in the nip angle α of the working rolls. As seen from the graph in Figure 7, with an increase in the values of the pulling force of conveyor chains *G*, the pressure of working rolls *Q* increases accordingly. Here, at lower values of *α*, the pressure of working rolls *Q* takes on more minor matters. Thus, with an increase in the importance of the nip angle and pulling force of conveyor chains *G*, the pressure of the working rolls *Q* increases accordingly.

**Figure 8:**
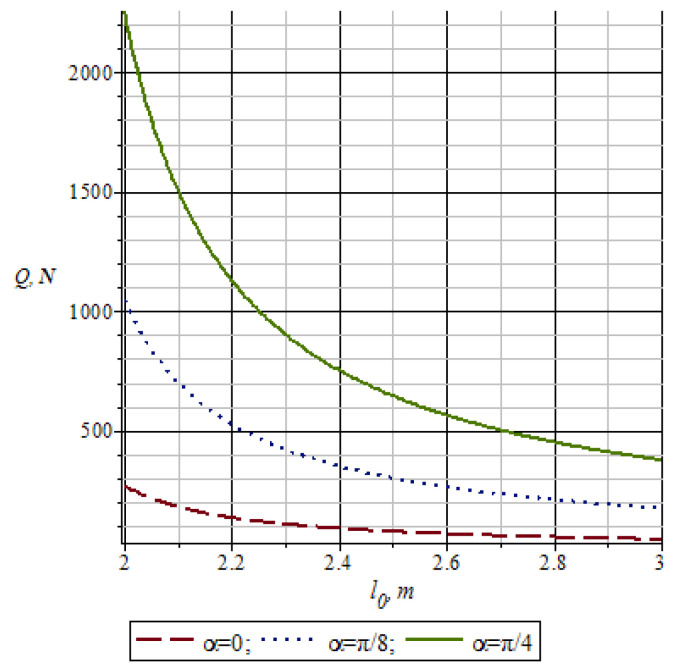
Graph of changes in the pressure of the working rolls depending on the length of the lever *l*_0_.

Figure 8 shows a graph of changes in the pressure of working rolls *Q* depending on the difference in the length of the small lever of the *L*-shaped lever 10 (Figure 5), taking into account the change in the nip angle *α* of the working rolls. From the graph in Figure 8, it can be seen that with an increase in the nip angle *α* of the working rolls and the length of the small lever of the *L*-shaped lever 10, the values of the pressure of working rolls *Q* decrease.

**Figure 9:**
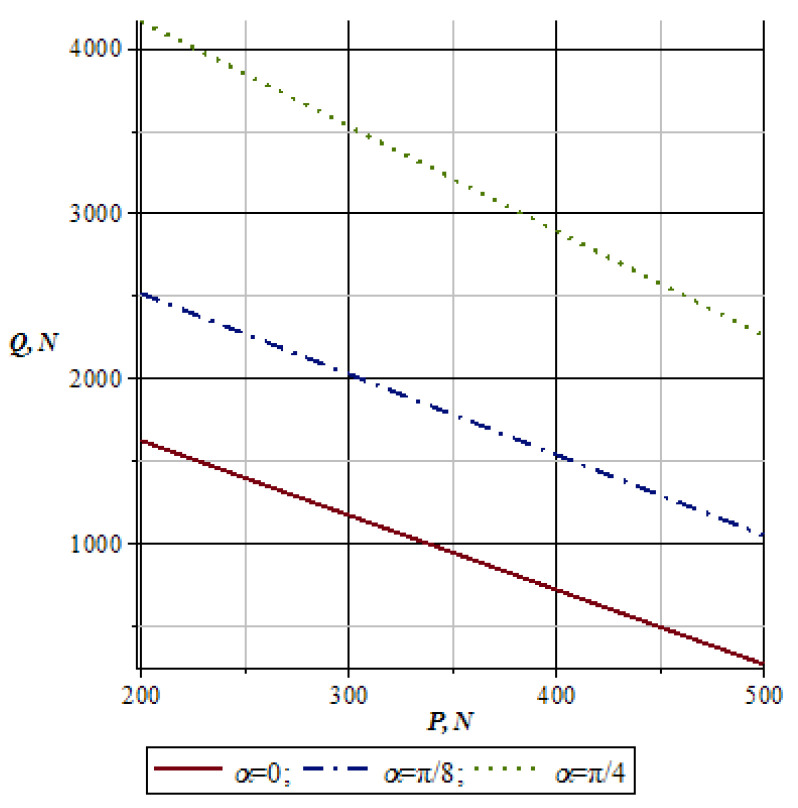
Graph of changes in the pressure of working rolls depending on their weight force.

Figure 9 shows the graph of changes in the pressure of working rolls *Q* depending on the difference in the weight force *P* of the material being processed, taking into account the change in the nip angle α of the working rolls.

From the graph in Figure 9, it can be seen that with an increase in the weight force *P* of the material being processed and the nip angle *α*, the pressure values of working rolls *Q* decrease accordingly. Thus, it will be necessary to increase the pressure of working rolls *Q* for greater values of weight force *P* of the material being processed.

**Figure 10:**
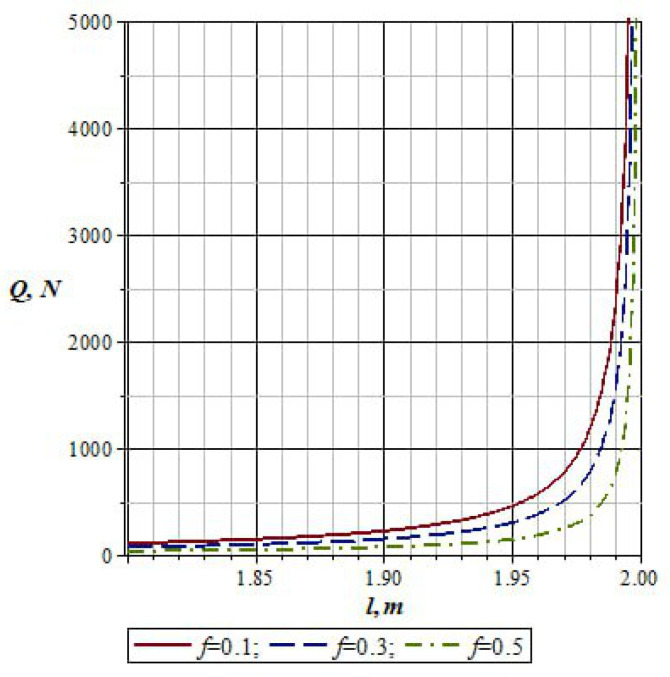
Graph of changes in the pressure of the working rolls depending on the length of the lever *l_2_*.

Figure 10 shows the graph of changes in the pressure of working rolls *Q* depending on the change in the length of the lever l2 of the *L*-shaped lever and weight *P* of the material being processed, taking into account the difference in the friction coefficient *f*_fr_. From the graph in Figure 10, it can be seen that with an increase in the length of the lever l2 of the *L*-shaped lever, the values of the pressure of the working rolls *Q* increase.

Here, with the value of friction coefficient *f*_fr_ = 0.1, the pressure of the working rolls increases, and for *f*_fr_ = 0.5, the pressure decreases.

**Figure 11:**
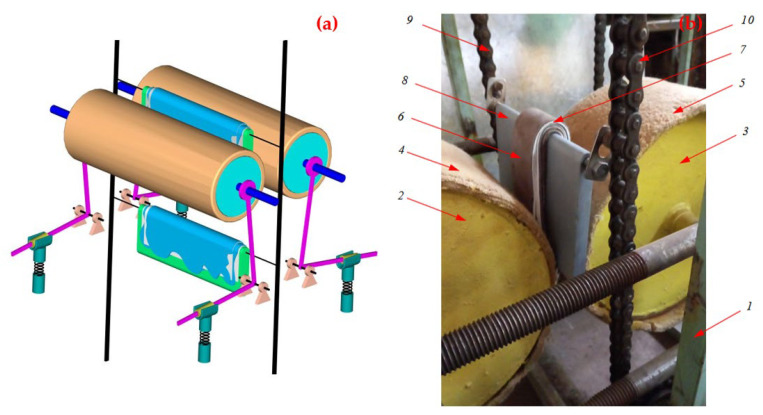
Schematic 3D view of a roller stand (**a**) and method for multi-layer dewatering of wet semi-finished leather products (**b**).1—stand frame, 2, 3—extrusion rollers, 4, 5—roller covers, 6—outer (first) layer of semi-finished product, 7—moisture-wicking fabric, 8—base plate, 9, 10—transport chains.

The authors have developed and produced an experimental roller stand for pressing multilayer leather semi-finished products (Figure 11). The 3D schematic view of a roller stand can be seen in Figure 11a. The squeezing rollers of this stand during processing shown in Figure 11b, namely upon contact with samples of semi-finished leather products, make a symmetrical displacement relative to each other along a horizontal line.

An experiment was conducted to determine the factors affecting the technological process of squeezing excess moisture from wet semi-finished leather products in a form of a multilayer package with moisture-removing material by means of their vertical supply on a base plate between rotating squeezing rollers, also covered with moisture-removing material. A critical analysis of the operation of the stand showed that a significant frontal impact occurred when the transported base plate came into contact with the squeezing rollers. Frontal resistance significantly affects the durability of the conveyor chains of the experimental stand. The transporting chains are subject to premature stretching. Therefore, in order to prevent undesirable stretching of the conveyor chains, we have developed and proposed the design of the mechanism for implementing the pressure of the squeezing rollers specifically for vertical-type roller machines. The pressure mechanism of the squeezing rollers is based on double-arm *L*-shaped levers. Such a design of the levers of the pressure mechanism allows the squeezing rollers to make a symmetrical arcuate displacement relative to each other when they come into contact with leather semi-finished products. This made it possible to reduce the frontal resistance exerted by the squeezing rollers on the transporting base plate. Consequently, this had a positive effect on the durability of the conveyor chains.

To determine the force that occurs during the contact of the processed sample with the squeezing shafts, we conducted an experiment in laboratory conditions between rotating shafts without their pressure. One layer of a semi-finished leather sample was passed through and measured using a lever system and a DPU-01-2 dynamometer (1000 N, “Tochpribor”, Moscow, Russia), and the resistance exerted by the squeezing rolls on the passed semi-finished leather sample was measured. The force was measured when the semi-finished leather layer on the transported base plate was touched by the rotating squeeze rollers. In the first case, the squeezing rolls, upon contact with the sample, moved from each other symmetrically horizontally (the existing technology); at this moment, the force was fixed on average 30 N (repeated 5 times). In the second case, the shafts, upon contact with the sample, moved from each other symmetrically along an arcuate trajectory (proposed technology); at this moment, the force was fixed at 15 N (repeated 5 times). Therefore, the measurement results of this experiment showed that the arcuate movement of the wringing shafts, as shown in Figure 2, significantly reduces drag, which is the purpose of this work.

In addition, due to the use of spherical rolling bearings on the axes of the squeezing shafts, as well as due to the installation of each axis on a separate double-arm L-shaped lever, it makes it possible to copy the unevenness of the machined (for example, in the form of a wedge). Here, the squeezing rolls change their position symmetrically, that is, at a certain angle (from 2° to 4°) depending on the thickness of the material being processed. At the same time, more force from the squeezing rolls falls on the thick section of the sample and the uniformity of residual moisture is ensured over the entire area of the sample. Before the start of the experiment, a moisture-removing cloth 0.008 m thick was placed in a kink on the transporting base plate, followed by a wet semi-finished leather product 0.004 m thick, and so forth; the total thickness was 0.024 m. The samples were weighed before and after squeezing on a FA2204C laboratory balance (WANT, Jiangsu, China) with a resolution of 0.0001 g (ISO-9001), as shown in Table 1.

When planning the experiment on the basis of a priori information, the process of moisture removal was studied taking into account two factors: *x*_1_ is the pressing intensity *P*, kN/m; *x*_2_ is the feed velocity *V*, m/s; the range of pressure change is from 32 to 96 kN/m; the speed of rotation of squeezing rollers is from 0.17 to 0.34 m/s; the number of layers of semi-finished leather products is 2. These parameters were selected based on the analysis of various analogs of squeezing technological machines. Figure 11a shows a schematic representation of a roller device; in the design of the pressure mechanism of the wringing rollers, elastic elements (springs) are used, which must be equipped with a hydraulic system. Figure 11b shows a fragment of the process of processing two layers of wet leather semi-finished products using moisture-removing felts between the wringing rolls.

Based on the results of the experiment, regression Equations (12) and (13) were obtained for two layers of leather semi-finished products. The resulting regression equations can be considered suitable with a 95% confidence probability, which, in a named form after decoding, has the following form:

For the first layer of semi-finished leather:

(12) $$\Delta W_{1}=23.93+0.00012119\cdot P^{2}-86.63\cdot V^{2}+0.23\cdot P+44.76\cdot V-0.36\cdot P\cdot V$$

For the second layer of semi-finished leather:

(13) $$\Delta W_{2}=29.7+0.001111\cdot P^{2}+46.75\cdot V^{2}+0.02\cdot P-44.02\cdot V-0.06\cdot P\cdot V$$

In this case, the residual moisture in two samples of semi-finished leather products had to be in the order of 60% out of the available 73% moisture. According to the requirement of the spinning technology, we needed to remove a maximum of 13% of the moisture during the spinning on the roller device.

It has been experimentally determined that with a roll pressing force of 32 kN/m (VOPM-1800-K, Gribanovsky Engineering Plant, Gribanovsky, Russia), the minimum removal was 22.2% for the first layer of the semi-finished leather product and 20.7% for the second layer. With a pressing force of the rolls of 64 kN/m (roller wringer devised by the Deri-Maksan Group, İzmir, Turkey), the minimum pressure for the first layer of the semi-finished leather product is 26.4% and for the second layer of the semi-finished leather product, it is 25.2%. Moreover, with the roll pressing force of 96 kN/m (Svit 07599/PЗ, Zlin, Czech Republic), the minimum moisture removal is 30.7% for the first layer of the semi-finished leather product and 25.2% for the second layer of the semi-finished leather product.

From the analysis of the data obtained from the experiment, the following conclusion was arrived at: the maximum difference between the amount of moisture removed from the first and second layers of semi-finished leather products is 3%. Thus, it can be stated that the technical requirements of the squeezing process are met.

Based on the results of the experiment, the optimal process parameters were selected. The process of squeezing the moisture from two layers of wet semi-finished leather products is recommended to be performed at a feeding speed of more than 0.34 m/s (two times faster than in analogs), with a pressing force of the squeezing rollers of 32 kN/m and more (two times lower than in analogs).

The experiment showed that the productivity of the technological process of squeezing wet semi-finished leather products increased by three times or more compared to the productivity of similar squeezing technological machines.

## 4. Conclusions

The developed device differs from existing ones in that the drive of the base plates with a package of wet leather semi-finished products and moisture-removing materials is realized via two conveyor chains and eight sprockets mounted on the bed using a gearbox shaft and an electric motor. The drive of the working rolls is recognized using sprockets and an endless chain around them, which are installed at the ends of the axes of the working rolls. The diameters of the working rolls are smaller than the diameters of the sprockets.

The authors offered an improved pressure mechanism of the working rolls for the squeezing machine. When implementing the technological process of pressing wet leather semi-finished products, depending on the quantity and thickness of the entire package, the working rolls move symmetrically along a specific arcuate trajectory. This will increase the durability of the conveyor chains of the roller machine.

Based on theoretical studies conducted to feed the semi-finished leather product between the squeezing rolls, graphs were plotted, and conclusions were drawn.

The initial parameters of the pressure mechanism of the roller machine were obtained. The change in the pressure parameters of the working rolls was determined using the equation of static equilibrium of forces. The dependencies of the pressure of the working rolls on the parameters of the pulling force of conveyor chains, gravity, nip angle, and friction coefficient were investigated and established.

Thus, it can be concluded that in the proposed improved pressure mechanism for the working shafts of a technological machine, the amount of deformation change will proportionally depend on the thickness of the layers of processed materials from the base plate together with moisture-removing materials. Consequently, there will be a regulation of the pressing force from the thickness of the entire package, which significantly improves the quality of the processing of squeezing the moisture from wet leather semi-finished products. This advantage of the proposed pressure mechanism is significantly reflected in multilayer moisture extraction from several layers of semi-finished leather products, since it will not necessarily regulate the constancy of the pressure of the working rollers, depending on the thickness of the processed package. Simultaneous processing of several layers of semi-finished leather products between rotating working shafts significantly increases the productivity of both the technological process and the roller technological machine.

It should be noted that on the above-mentioned roller wringers, raw hides are processed with only one layer. According to the technology we proposed, it was possible to carry out the extraction of wet semi-finished leather products in two or more layers. Consequently, the productivity of the processing process according to the proposed technology is significantly increased (in this case, two times or more) compared to the above roller wringers. Thus, a simplified and simultaneously effective design of a roller technological machine is proposed, which allows the simultaneous processing of wet semi-finished leather products in several layers (in a package) and thereby increases the productivity of the spinning process depending on the number of processed layers.

## Figures and Tables

**Table 1:** Experimental processing results.

№	*P*, x_1_	*V*, x_2_	Sample Number	Measurement Results, (%)						$\sum_{1}^{n}{\left(y-\bar{y}\right)}^{2}$	$S_{er}^{2}$	$y_{cal}$	$\bar{y}-y_{cal}$	${\left(\bar{y}-y_{cal}\right)}^{2}$
				*y*_1_	*y*_2_	*y*_3_	*y*_4_	*y*_5_	$\bar{y}$					
1	0	0	1	30.4	24.8	24.7	24.3	27.6	26.9	24.55	6.14	27.4	0.5	0.25
			2	20.7	24.4	24.7	35.7	23.7	25.7	101.85	25.46	26.0	0.3	0.09
2	+	+	1	30.0	33.3	29.8	23.9	36.4	30.7	86.79	21.70	29.0	1.7	2.87
			2	31.2	27.2	25.4	33.9	30.5	29.6	43.06	10.77	29.7	0.1	0.01
3	−	+	1	26.7	16.9	24.7	20.6	21.9	22.2	57.24	14.31	21.1	1.1	1.21
			2	20.6	16.6	27.2	20.5	18.8	20.7	62.72	15.68	16.80	0	0
4	−	−	1	24.6	28.7	25.4	22.5	25.5	25.3	19.94	4.99	23.9	1.4	1.96
			2	25.2	23.0	25.7	26.9	23.8	24.9	9.55	2.39	24.8	0.1	0.01
5	+	−	1	31.2	37.8	36.8	33.5	39.1	36.7	47.47	11.87	34.8	1.9	3.61
			2	34.4	33.9	33.2	39.4	31.6	34.5	32.88	8.22	34.24	0.3	0.09
6	+	0	1	32.2	28.2	35.4	36.9	29.8	32.5	53.64	13.41	32.5	0	0
			2	28.1	25.8	29.8	23.9	36.4	31.4	126.06	31.52	31.6	0.2	0.4
7	0	+	1	24.0	24.4	21.7	35.7	26.2	26.4	118.38	29.60	26.2	0.2	0.04
			2	25.0	25.9	23.5	26.7	24.9	25.2	5.76	1.44	24.3	0.1	0.01
8	−	0	1	22.2	21.4	34.0	21.4	24.8	24.7	114.53	28.63	22.6	2.1	4.41
			2	18.2	24.6	18.0	30.7	24.5	23.2	111.94	27.99	22.6	0.6	0.36
9	0	−	1	27.8	27.0	38.7	28.0	25.1	29.3	113.23	28.31	28.7	0.6	0.36
			2	26.8	31.4	28.5	27.4	24.8	27.8	23.61	5.90	28.4	0.6	0.36
			1							∑635.77	∑158.94			∑14.71
			2							∑546.27	∑136.57			∑0.97
